# Somatic mutations in benign breast disease tissues and association with breast cancer risk

**DOI:** 10.1186/s12920-021-01032-8

**Published:** 2021-07-14

**Authors:** Stacey J. Winham, Chen Wang, Ethan P. Heinzen, Aditya Bhagwate, Yuanhang Liu, Samantha J. McDonough, Melody L. Stallings-Mann, Marlene H. Frost, Robert A. Vierkant, Lori A. Denison, Jodi M. Carter, Mark E. Sherman, Derek C. Radisky, Amy C. Degnim, Julie M. Cunningham

**Affiliations:** 1 Biomedical Statistics and Informatics, Mayo Clinic, 200 First Street SW, Rochester, MN 55905 USA; 2 Medical Genome Facility, Mayo Clinic, 200 First Street SW, Rochester, MN 55905 USA; 3 Cancer Biology, Mayo Clinic, 4500 San Pablo Road South, Jacksonville, FL 32224 USA; 4 Women’s Cancer Program, Mayo Clinic, 200 First Street SW, Rochester, MN 55905 USA; 5 Information Technology, Mayo Clinic, 200 First Street SW, Rochester, MN 55905 USA; 6 Laboratory Medicine and Pathology, Mayo Clinic, 200 First Street SW, Rochester, MN 55905 USA; 7 Epidemiology and Laboratory Medicine and Pathology, Mayo Clinic, 4500 San Pablo Road South, Jacksonville, FL 32224 USA; 8 Breast, Endocrine, Metabolic and GI Surgery, Mayo Clinic, 200 First Street SW, Rochester, MN 55905 USA; 9 Experimental Pathology and Laboratory Medicine, Mayo Clinic, 200 First Street SW, Rochester, MN 55905 USA

**Keywords:** Breast cancer risk, Benign breast disease, Somatic mutations, Mutation burden, CD45 expression

## Abstract

**Background:**

Benign breast disease (BBD) is a risk factor for breast cancer (BC); however, little is known about the genetic alterations present at the time of BBD diagnosis and how these relate to risk of incident BC.

**Methods:**

A subset of a long-term BBD cohort was selected to examine DNA variation across three BBD groups (42 future estrogen receptor-positive (ER+) BC, 36 future estrogen receptor-negative (ER−) BC, and 42 controls cancer-free for at least 16 years post-BBD). DNA extracted from archival formalin fixed, paraffin-embedded (FFPE) tissue blocks was analyzed for presence of DNA alterations using a targeted panel of 93 BC-associated genes. To address artifacts frequently observed in FFPE tissues (e.g., C>T changes), we applied three filtering strategies based on alternative allele frequencies and nucleotide substitution context. Gene-level associations were performed using two types of burden tests and adjusted for clinical and technical covariates.

**Results:**

After filtering, the variant frequency of SNPs in our sample was highly consistent with population allele frequencies reported in 1 KG/ExAC (0.986, p < 1e−16). The top ten genes found to be nominally associated with later cancer status by four of 12 association methods(p < 0.05) were *MED12, MSH2, BRIP1, PMS1, GATA3*, *MUC16, FAM175A, EXT2, MLH1* and *TGFB1*, although these were not statistically significant in permutation testing. However, all 10 gene-level associations had OR < 1 with lower mutation burden in controls compared to cases, which was marginally statistically significant in permutation testing (p = 0.04). Comparing between the three case groups, BBD ER+ cases were closer to controls in mutation profile, while BBD ER− cases were distinct. Notably, the variant burden was significantly higher in controls than in either ER+ or ER− cases. CD45 expression was associated with mutational burden (p < 0.001).

**Conclusions:**

Somatic mutations were more frequent in benign breast tissue from women who did not develop cancer, opening questions of clonal diversity or immune-mediated restraint on future cancer development. CD45 expression was positively associated with mutational burden, most strongly in controls. Further studies in both normal and premalignant tissues are needed to better understand the role of somatic gene mutations and their contribution to future cancer development.

**Supplementary Information:**

The online version contains supplementary material available at 10.1186/s12920-021-01032-8.

## Background

Breast cancer (BC) is a leading cause of cancer in women, and is believed to result from the progressive accumulation of genetic and epigenetic alterations, where genomic analyses have revealed both inherited predisposition and common genetic variation play a role in susceptibility [1–3]. However, the majority of women with BC have no major germline mutations and thus develop sporadic cancers, raising questions. Early detection is critical to detect and prevent cancer-related death, as early-stage BC has a 99% five-year survival rate, compared to 27% for advanced stage cancers [4]. Identification of biomarkers that predict progression in cancer precursors could enable improved management, where high-risk women are offered closer surveillance and preventative treatments and low-risk women may be screened less frequently. Benign breast disease (BBD), which includes non-proliferative, proliferative and proliferative lesions with atypia, is viewed as a nonobligate precursor stage in the development of BC [5], and is associated with an increased risk of invasive BC, particularly in those with proliferative or atypical lesions [6–8].

As the majority of women with BC have no major germline mutations and thus develop sporadic cancer [9, 10], the platform of benign breast disease offers a window in early carcinogenetic events. Questions remain regarding the processes that drive deoxyribonucleic acid (DNA) mutations in breast tissue for patients with sporadic BC, and which mutations are associated with the earliest stages of BC development. In particular, atypical hyperplasia has been reported to share genomic changes with common sporadic BC, including structural genomic changes such as aneuploidy, loss of heterozygosity, and large-scale amplifications and deletions [11, 12]. In addition to structural alterations, analysis of DNA mutations present in BBD biopsies, which contain both the BBD lesion and the surrounding tissue bed, has the potential to define the processes that drive the development of cancer-associated DNA mutations, as well as which of those mutations are most critical for cancer development [11, 12].

To date, few DNA sequencing studies have examined mutational status in BBD tissues. Rohan et al. [13] sequenced 218 BBD cases that subsequently developed invasive BC and matched cancer free BBD controls using a targeted capture-based panel and reported no significant mutation burden differences. Adjacent, non-malignant tissue was used as a surrogate for germline variants which were excluded. Soysal et al. [14] used a targeted amplicon-based sequencing panel to profile 17 cases of invasive BC with a previous diagnosis of fibrocystic disease, all with matching normal tissue. They reported that no significant mutations in hotspot residues were seen in either tumor or benign disease. A recent report by Zeng et al. [15] detailed whole exome sequencing on 135 BBD cases that subsequently developed cancer and 69 cancer free controls, using a subset of patients with available germline DNA and a neural network to predict somatic variants in the unrelated BBD samples. While finding no significant difference in the number of mutations between cases and controls, when filtering to variants with variant allele frequency (VAF) > 25%, non-silent mutation differences were observed between cases and controls for some but not all genes detected at lower VAF.

The Mayo Clinic BBD cohort was first described in 2005 [5], and includes more than 13,258 BBD cases with a median follow-up of 13 years of clinical data [16]. To identify underlying genetic aberrations in BBD cases associated with future BC occurrences, we designed a DNA sequencing study of 120 patients with formalin-fixed paraffin-embedded (FFPE) BBD tissues. This study focused on three groups, those patients who remained cancer-free after at least 16 years post-BBD (controls), and those developing estrogen receptor positive (ER+) or estrogen receptor negative (ER−) invasive BC cancers within 16 years. Analysis focused on comparison of controls to the cancer groups, and between ER+ and ER− cancers.

## Methods

### Cohort constructions

The Mayo Clinic Benign Breast Disease (BBD) Cohort includes 13,455 women, ages 18–85 who underwent benign biopsies at Mayo Clinic between 1967 and 2001. Women who had been diagnosed with invasive or in situ BC before or within six months of biopsy or have undergone risk-reducing mastectomy or breast reduction surgery prior to biopsy were excluded. Among this cohort, a frequency-matched (by age and year of biopsy) case–control sample was selected, where cases were defined as those women with BBD who subsequently went on to develop either ER+ or ER− BC within 16 years, and controls were defined as women with BBD who had not developed BC after at least 16 years of follow-up. Index benign biopsies were screened from women with ER− or ER+ BC and corresponding controls, matched on age at biopsy, year of biopsy, and length of follow-up time/time to BC diagnosis. After determining tissue block availability, adequate DNA amount and quality, and adequate sequencing quality, our final sample set for association analysis included 42 ER+ cases, 36 ER− cases, and 42 controls diagnosed between 1969 and 2001. Demographic and clinical characteristics were compared across groups using Pearson chi-square tests for categorical variables and ANOVA tests for continuous variables.

### DNA extraction and sequencing

DNA extraction and sequencing were performed as previously reported [17]. In brief, DNA was extracted from ten micrometer sections of FFPE or fresh frozen tissue using the GeneRead DNA FFPE kit (Qiagen, Germantown, MD, US). After extraction, DNA was quantified using Qubit™ dsDNA BR Assay (ThermoFisher Scientific, Waltham, MA, USA) while quality was assessed using the Advanced Analytical Fragment Analyzer™ High Sensitivity Large Fragment Analysis kit which calculates fragment length and degradation.

The QIAseq Human Breast Cancer Targeted Panel, which targets 93 genes relevant in BC, was used to create libraries using 20–40 ng of DNA as previously reported [17], following Qiagen guidelines for FFPE DNA. Libraries were quantified and sequenced on an Illumina® HiSeq 4000 (Illumina, San Diego, CA, USA) paired end 150-bp.

To facilitate quality control and to provide confidence in results derived from archival FFPE tissue, a set of technical control samples was used, and included four fresh snap-frozen benign breast samples (with pathologic assessment of cryoH&E sections) and paired FFPE tissues from reduction mammoplasties, one CEPH control NA12891 (Coriell Institute for Medical Research, Camden, NJ, USA), one FFPE BBD sample not in the sample set, and a positive control sample from formalin fixed cell lines with 11 mutations at varying allelic frequencies (Horizon Diagnostics LLC, Columbus, GA, USA). Variants were located within the *BRAF*, *cKIT*, *EGFR*, *KRAS*, *NRAS* and *PIK3CA* genes.

### DNA-seq alignment and variant calling

The Qiagen Data Portal was used for primary sequencing analysis of the samples [18]. The analysis steps included adapter trimming, coupling molecular tag (MT) sequence to the read IDs, alignment to the reference genome (GRCh37 build), and subsequent variant calling using smCounter, a molecular tag-aware variant calling algorithm. smCounter uses a Bayesian probabilistic model to identify variants and infer genotypes, which has been shown to detect low frequency variants with high sensitivity [19]. Sequenced reads with identical molecular tags were identified as PCR duplicates. Reads identified as PCR duplicates were collapsed to create a consensus read sequence. A molecular diversity score, defined as the proportion of molecular-tag coverage versus raw sequencing coverage (100 × MT-coverage/Raw-coverage), was calculated for each sample. For variant calling, the consensus sequence was compared to the reference genome and a prediction index of the alleles observed at the molecular tag level was calculated for every target position. A variant was called if the prediction index of an allele was higher than the pre-specified prediction index threshold, based on 8 reads per molecular tag [19]. The resulting variant calls were output in the standard VCF. After variant calling, initial variant filtering excluded likely false calls due to technical factors, including shallow molecular tag coverage, strand bias, presence in low complexity regions, and/or low base quality.

### Sample QC and acceptance criteria

Samples with an average unique molecular tag (UMT) coverage of < 20 × and with genotyping call rate of SNPs < 80% were excluded. For each sample, variants were called as genotypes based on bins of allele frequencies of 0–0.2 (rare homozygote), 0.4–0.6 (heterozygote), and 0.8–1.0 (common homozygote), and the genotyping call rate was defined as the proportion of SNPs for which the genotype was called. The distribution of genotyping call-rate versus the mean UMT coverage is shown in Additional File 1: Fig. S1.

Sample identity was examined using Spearman correlation of the minor allele frequency of known SNPs across all samples to identify those emanating from the same individual. For quality control, 24 samples with two to 10 replicates were profiled independently, with a total number of 119 replicate-pairs. After strict variant filtering, correlation values of replicates were completely separable from unrelated samples; replicate samples had correlation close to 1.0 (all > 0.85), whereas unrelated samples had correlation centered around 0.6 (all < 0.85) (see Additional File 2: Fig. S2; Metadata on the final sample set are presented in Additional File 3: Table S1).

### Additional variant filtering and population allele frequency concordance

As FFPE samples are known to be prone to variant artifacts, additional filtering was required before further analysis. Utilizing paired FFPE and fresh frozen samples that we studied previously with identical methods [17], false discovery rate (FDR) of variant-calling was approximated for seven mutation types (C>A, C>G, C>T, C > T at CpG, T > A, T>C and T>G) (see Additional File 4: Fig. S3). The empirical relationships between alternate allele frequency (AAF) and FDR were utilized to determine further filtering strategies. We defined three sets of variants based on liberal, classical or strict filtering of AAF. For the liberal set, variants with AAF < 0.05 were removed. For the classical set, C>T mutations (with the exception of SNPs annotated with an rsID) with AAF < 0.1 were removed, while other types of mutations with AAF < 0.05 were removed. For the strict set, all variants with AAF < 0.1 were removed.

After additional AAF-based filtering, variant frequencies in our cohort were compared with population allele frequencies to ensure that additional filtering strategies did not skew allele distribution of study samples. In particular, all the detected variants were annotated with population allele frequencies observed in the 1000 Genomes Project and the Exome Aggregation Consortium (ExAC) based on internally developed bioR annotation software [20], and were compared between the overlapping variants.

### Gene-level association methods

To prioritize variants for association analyses, those meeting any of the following criteria were removed: 1. Observed in common populations with minor allele frequency (MAF) > 0.5%, according to Genome Aggregation Database (gnomAD) and Trans-Omics for Precision Medicine (TOPMED) studies [21, 22]. 2. A small number of variants (N = 44) variants that were common in BBD controls (average VMF over the controls > 0.05). 3. Defined as low functional impact by the CAVA bioinformatics annotation tool [23]. In the CAVA annotation process, medium and high impact variants were defined as essential splice bases, stop gain, frameshift, nonsynonymous variant, inframe indels, start codon, stop loss, and/or exon end (alters first or last three bases of exon).

The AAFs were summarized at each position, overall, and by group (ER+, ER−, control). Frequencies were also summarized for each gene and overall, across genes.

Gene-level analyses considered mutations in two ways: (1) as a continuous allele frequency, and (2) as a binary presence/absence of mutation variable. Gene-level analyses used logistic regression to compare the sum of continuous variant allele frequencies across variants in cases (all cases, as well as separately in ER+ and ER−) versus controls. Additionally, gene-level analyses of the binary presence/absence of mutations across a gene were conducted with SKAT-O. In addition to the default weighting based on variant allele frequencies, secondary analyses also implemented a more stringent variant weighting where each mutation was down-weighted according to its FDR, and hence C->T mutations were more heavily down-weighted compared to other mutation types. This resulted in four statistical analysis methods: SKAT-O, C-T down-weighted SKAT-O, logistic regression, and C-T down-weighted logistic regression; when combined with the three levels of variant AAF filtering (liberal, classic, and strict, defined above), this yielded 12 combinations of statistical method and filtering criteria.

All models were adjusted for relevant covariates, including epithelial percentage, histologic impression, patient’s age, year of biopsy (including a linear term for year, indicator for whether the biopsy was post-1992, year*1992 interaction due to an FFPE processing change), and SNP call rate. Sensitivity analyses were performed for the different variant QC criteria (strict, classical, and liberal). Primary analyses compared all cases (ER+ and ER−) to controls, but secondary analyses considered ER+ and ER− cases separately.

In order to assess statistical significance of the gene-level results, permutation tests were conducted. The sample labels were permuted 100 times (with the relationship between the covariates and group status preserved), and the gene-level analyses across the 12 combinations of variant filtering and statistical analysis method were performed. Empirical p-values were calculated from the distribution of two quantities: (1) the number of genes with p-value < 0.05 in 4 out of 12 methods (two-sided test) and (2) the number of genes with p-value < 0.05 and OR < 1 under the weighted logistic regression model with classical variant filtering (one-sided test).

### Mutational signature analysis

Using the high-impact variants with classic AAF filtering, a set of custom Perl scripts was used to generate a mutation frequency table of all SNVs across each sample to assess the of mutation types being reported. This mutation frequency table was subsequently used to generate plots of mutation signatures for the variants using Perl and R scripts (code available from GitHub repository https://github.com/Liuy12/BBD_generead). Mutational spectrums and *de-novo* mutational signatures were identified using the MutationalPatterns package (version 1.2.1) [24]. *De-novo* signatures were extracted based on a non-negative matrix factorization (NMF) algorithm. Through consensus clustering, four stable *de-novo* mutational signatures were identified and compared with COSMIC mutational signatures v2 (n = 30) based on cosine similarity (Additional File 5: Fig. S4) [25]. Signature A (QGR signature) matches the patterns identified in samples processed with the FFPE DNA protocol used, the QIAGEN GeneRead DNA FFPE Kit ("QGR") [17]. Signature B (FFPE signature) matches patterns observed in FFPE samples but not in fresh-frozen samples. Signature C (Block year signature) was highly associated with the year of FFPE block creation. Signature D (Residual signature) had no clear correspondence with previously found mutational signatures. The estimated signatures are shown in Additional File 6: Table S2.

### Immunohistochemistry analysis

In all BBD samples, expression of Ki67 and CD45 was assessed in up to 10 normal lobules using IHC. Immunostaining was performed using the following antibodies: CD45 (Abcam ab10559, 1:800), Ki67 (DAKO M7240, 1:100). Samples were deparaffinized with three changes of xylene, rehydrated in 95% ethanol and rinsed well in running distilled water. Slides were then placed in a preheated Antigen Retrieval solution (pH 6.0, DAKO) for 25 min and then cooled in the buffer for 25 min followed by a five-minute rinse in running distilled water. After the heat-inactivated epitope retrieval step, slides were placed on the DAKO Autostainer at room temperature for the following procedure. Sections were incubated with 3% H_2_O_2_ for five minutes to inactivate the endogenous peroxides and then incubated in the primary antibody at dilutions listed above for 60 min at room temperature. Sections were rinsed with Tris-buffered saline/Triton-X100 (TBST) wash buffer and incubated with the secondary antibody (Envision (+) anti-mouse labeled polymer (HRP, K4001) for Ki67 and Envision (+) anti-rabbit labeled polymer (HRP, K4003) for CD45) for 30 min. Slides were then rinsed with TBST wash buffer and sections were incubated in 3,3′-diaminobenzidine (DAB+) (K3467, DAKO) for five minutes, counterstained with Gills I hematoxylin for one minute, followed by a three-minute tap water rinse to blue sections, dehydrated through graded alcohols and cleared in three changes of xylene and mounted with permanent mounting media. Slides were scanned using the Aperio™ Scanscope XT Slide Scanner (Leica Biosystems, Buffalo Grove, IL, USA) with image acquisition and staining quantitation using Aperio™ ImageScope Software (Leica Biosystems, Buffalo Grove, IL, USA). Slides were scanned at resolution of 0.5 µm/pixel with no downstream image processing. Ki67 was measured as percent positive nuclei and CD45 was measured as an H score (a combined measure of the intensity and extent of staining) [26–28]. CD45 and Ki67 were assessed as continuous measures. Associations across groups were evaluated as Wilcoxon rank-sum tests, and associations with mutational burden (based on classical variant filtering) were evaluated with Spearman correlation.

## Results

### Sample characteristics

The clinicopathologic characteristics of the samples selected for this study are shown in Table 1. Among the full BBD Cohort, a frequency-matched case–control sample set of BBD biopsies was selected based on outcome in follow-up at 16 years: incident ER + BC (BBD-ER+), incident ER− BC (BBD-ER−) or cancer-free (BBD-control), matched on age at biopsy and year of biopsy or censoring. Selection criteria also included availability of blocks with adequate tissue for DNA extraction. Severity of BBD was the only feature that differed significantly among the three groups (p = 0.026). The BBD-controls included the highest percentage of non-proliferative disease (n = 25; 59.5%), while the BBD-ER− group had the highest proportion of proliferative disease without atypia (n = 19; 52.8%) and the BBD-ER+ group had the highest proportion of atypical hyperplasia (n = 9; 21.4%), consistent with previous studies [5].

**Table 1:** Cohort characteristics

	Cancer free at 16 years (N = 42)	ER negative BC (N = 36)	ER positive BC (N = 42)	Total (N = 120)	P value*
Age					0.416
< 45	11 (26.2%)	11 (30.6%)	7 (16.7%)	29 (24.2%)	
45–55	19 (45.2%)	14 (38.9%)	16 (38.1%)	49 (40.8%)	
> 55	12 (28.6%)	11 (30.6%)	19 (45.2%)	42 (35.0%)	
Histologic impression					0.026
Non-proliferative disease	25 (59.5%)	14 (38.9%)	19 (45.2%)	58 (48.3%)	
Proliferative disease without Atypia	16 (38.1%)	19 (52.8%)	14 (33.3%)	49 (40.8%)	
Atypical hyperplasia	1 (2.4%)	3 (8.3%)	9 (21.4%)	13 (10.8%)	
Atrophy					0.086
N-miss	2	3	3	8	
None	9 (22.5%)	13 (39.4%)	6 (15.4%)	28 (25.0%)	
Partial	16 (40.0%)	13 (39.4%)	23 (59.0%)	52 (46.4%)	
Complete	15 (37.5%)	7 (21.2%)	10 (25.6%)	32 (28.6%)	
Year of BBD					0.452
Mean (SD)	1986 (8)	1987 (8)	1988 (9)	1987 (8)	
Range	1969–1996	1970–1999	1972–2001	1969–2001	

^*^Statistical comparisons for demographic variables across BBD group were conducted with Pearson’s chi-square test for categorical variables and ANOVA test for continuous variables

### Gene-level associations

**Fig. 1:**
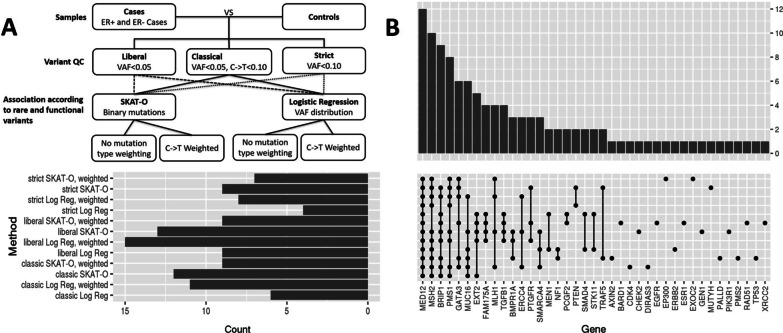
Gene-level association findings between cases (BBD with future cancer events) and controls (BBD without future cancer events up to 16 years): **a** analytical flows of 12 association and filtering methods. **b** Histogram with connected dot-plot summarizing consensus of significant genes detected by 12 methods

To address potentially artefactual FFPE variants, 12 combinations of variant filtering strategies and statistical analysis methods (classical, liberal and strict variant quality control (QC) filtering, combined with C-T weighted and un-weighted SKAT-O and logistic regression, described in Methods) were used to identify significant gene-level mutation burden differences between cancer (BBD-ER+ and BBD-ER−) and cancer-free (BBD-control) groups (Fig. 1a; Table 2). Full results are shown in Additional files 7–9: Tables S3–S5. No gene-level results were statistically significant after Bonferroni correction for 93 genes (p < 0.00054). Through consensus analysis of association results shown as Fig. 1b, 10 genes (*MED12, MSH2, BRIP1, PMS1, GATA3*, *MUC16, FAM175A, EXT2, MLH1* and *TGFB1)* of nominal significance (p < 0.05) were found according to at least four methods. Based on the results of the permutation tests, the empirical p-value from observing 10 or more genes with nominal significance in four out of 12 methods is 0.15.

**Table 2:** Association results of leading genes (top 10 genes, with P < 0.05 in 4 out of 12 methods)

Gene	SKAT-O p	SKAT-O p, weighted	Logistic Regression OR	Logistic Regression p	Logistic Regression OR, weighted	Logistic Regression p, weighted	Analysis
MED12	0.0475*	0.0390*	0.9239 (0.8455, 0.9916)	0.0268*	0.8138 (0.66, 0.9681)	0.0189*	Classic
	0.0067**	0.0016**	0.9472 (0.8917, 0.9941)	0.0261*	0.8441 (0.7129, 0.9696)	0.0148*	Liberal
	0.0445*	0.0342*	0.9205 (0.8368, 0.9923)	0.0293*	0.8326 (0.686, 0.9721)	0.0189*	Strict
MSH2	0.0203*	0.0445*	0.7539 (0.5546, 0.9418)	0.0078**	0.4294 (0.1775, 0.8518)	0.0085**	Classic
	0.0544	0.0856	0.8651 (0.7189, 0.9902)	0.0339*	0.618 (0.3305, 0.9584)	0.0277*	Liberal
	0.0284*	0.0145*	0.6938 (0.4566, 0.9331)	0.0097**	0.3202 (0.1025, 0.7632)	0.0043**	Strict
BRIP1	0.0252*	0.1015	0.8686 (0.7356, 0.9874)	0.0301*	0.7081 (0.4852, 0.9707)	0.0313*	Classic
	0.0087**	0.0319*	0.8873 (0.7758, 0.9777)	0.0131*	0.7271 (0.5182, 0.9436)	0.0146*	Liberal
	0.0468*	0.0809	0.864 (0.7049, 1.002)	0.0542	0.7046 (0.4418, 0.9956)	0.0469*	Strict
PMS1	0.0519	0.0935	0.8117 (0.6799, 0.949)	0.0081**	0.6126 (0.4005, 0.8929)	0.0101*	Classic
	0.0684	0.0842	0.8595 (0.745, 0.9704)	0.0134*	0.6628 (0.4569, 0.9149)	0.0116*	Liberal
	0.0165*	0.0136*	0.7846 (0.6447, 0.9309)	0.0047**	0.6053 (0.3996, 0.865)	0.0049**	Strict
GATA3	0.0046**	0.2446	0.7949 (0.5809, 1.004)	0.0544	0.4899 (0.2246, 0.938)	0.0304*	Classic
	0.0130*	0.4957	0.8798 (0.7154, 1.043)	0.1439	0.6355 (0.3493, 1.059)	0.0832	Liberal
	0.0051**	0.0175*	0.8086 (0.5848, 1.028)	0.0861	0.4766 (0.2047, 0.912)	0.0233*	Strict
MUC16	0.0151*	0.0021**	0.9875 (0.9726, 0.999)	0.0327*	0.9588 (0.9153, 0.9927)	0.0157*	Classic
	0.1559	0.1089	0.9934 (0.9847, 1.001)	0.0746	0.9767 (0.9494, 0.9992)	0.0424*	Liberal
	0.1247	0.0875	0.988 (0.9723, 1)	0.0586	0.9664 (0.9259, 0.9982)	0.0376*	Strict
EXT2	0.1100	0.1604	0.8305 (0.6589, 0.997)	0.0461*	0.5994 (0.3212, 1.01)	0.0549	Classic
	0.0404*	0.0494*	0.8593 (0.72, 0.9822)	0.0244*	0.6348 (0.3789, 0.9543)	0.0277*	Liberal
	0.1435	0.1836	0.8292 (0.6447, 1.009)	0.0620	0.6434 (0.3599, 1.038)	0.0718	Strict
FAM175A	0.0657	0.1724	0.7249 (0.4568, 1.014)	0.0612	0.3819 (0.09252, 1.061)	0.0669	Classic
	0.0103*	0.0181*	0.7172 (0.5108, 0.9316)	0.0095**	0.3513 (0.1179, 0.8153)	0.0114*	Liberal
	0.1628	0.1461	0.7465 (0.4696, 1.05)	0.0981	0.4276 (0.1126, 1.089)	0.0793	Strict
MLH1	0.1012	0.0885	0.8551 (0.7103, 1.005)	0.0573	0.6972 (0.4748, 0.9608)	0.0267*	Classic
	0.1320	0.0929	0.8819 (0.7573, 1.002)	0.0529	0.7201 (0.5073, 0.9604)	0.0244*	Liberal
	0.0895	0.0168*	0.8393 (0.672, 1.021)	0.0801	0.7335 (0.511, 0.9976)	0.0482*	Strict
TGFB1	0.1689	0.1067	0.7878 (0.542, 1.077)	0.1381	0.5017 (0.1886, 1.029)	0.0610	Classic
	0.0070**	0.0146*	0.7198 (0.5139, 0.9364)	0.0118*	0.4107 (0.1599, 0.8326)	0.0097**	Liberal
	0.6571	0.6260	0.8737 (0.5926, 1.236)	0.4487	0.6909 (0.2797, 1.412)	0.3246	Strict

P values less than two thresholds: p < 0.05 (*), p < 0.01 (**)

**Fig. 2:**
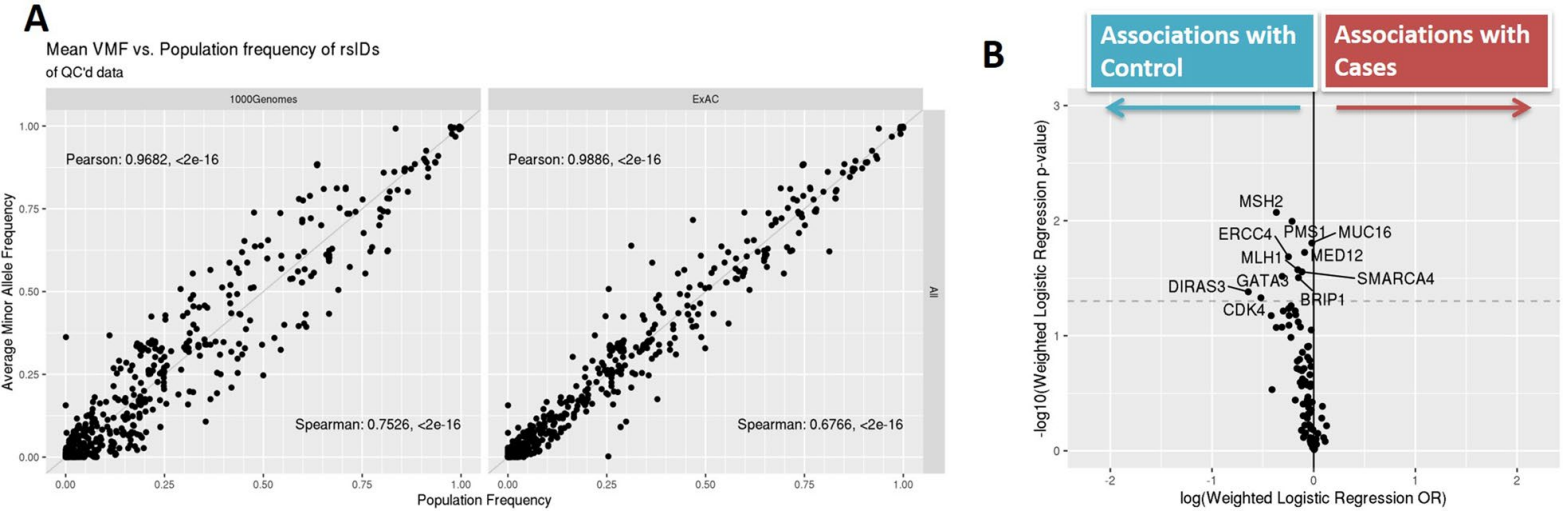
Variant concordances with normal genetics finding and gene-level volcano plots: **a** population frequency’s variant-level (x-axis) concordances with observed allele frequencies in this BBD cohort (y-axis). **b** Volcano plots of weighted logistic regression-based odds-ratio (OR) and statistical significance, for all the cases versus controls, using the classic definition of AAF variant filtering

After extensive sample- and variant-level quality control, common single nucleotide polymorphisms (SNP) variants detected in this cohort had highly consistent allele frequency distributions when compared with population frequencies derived from large-scale germline sequencing studies such as 1000 Genome Project and Exome Aggregation Consortium (ExAC), shown as Fig. 2a. The concordance of detected allele frequencies with population frequencies were persistent even when variants were stratified by nucleotide substitution type (see Additional File 10: Fig. S5). This strong concordance at the population-level suggests a solid basis for the association analyses. When comparing overall BBD cancer cases (BBD-ER+ and BBD-ER−) versus BBD-controls, a volcano plot of gene-level association effect-sizes by the corresponding significance levels showed a skewed distribution, with more significant findings enriched for more mutations in cancer-free subjects (Fig. 2b). Based on the results of the permutation tests, the empirical p-value for observing 10 or more genes with nominal significance and OR < 1 was 0.04; hence, the increased mutational burden in controls is marginally statistically significant. By further stratifying association analysis by type of BC (i.e. ER+ and ER−), the association differences were more profound when comparing BBD controls with BBD subjects with future ER− cancers than those with ER+ cancers, while the volcano plots remained skewed towards enrichment of protective associations (see Additional File 11: Fig. S6).

### Mutational signatures

**Fig. 3:**
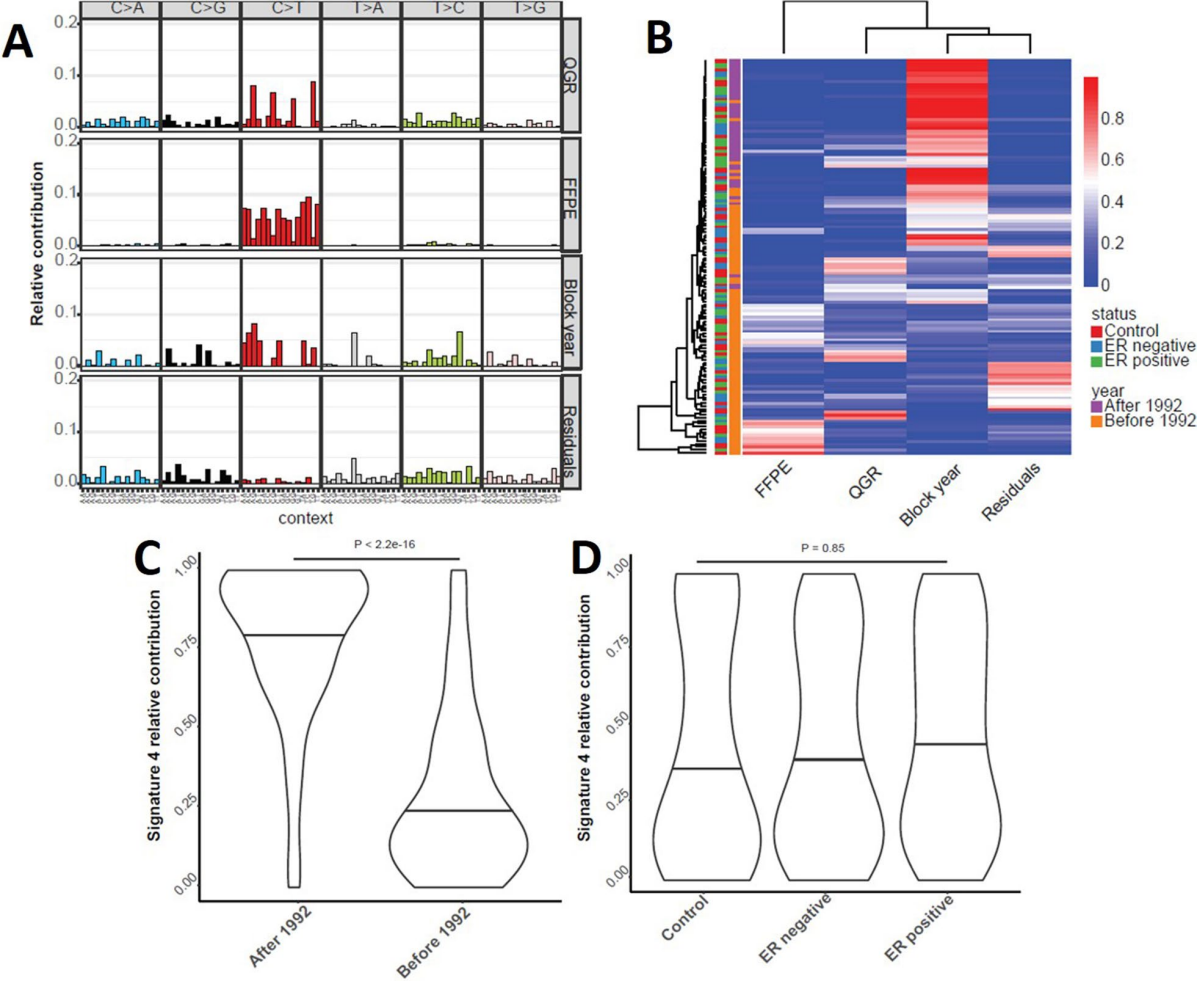
*De-novo* mutational signatures of entire dataset: **a** four dinucleotide signatures found through NMF under the classic AAF filtering definition. **b** heatmap of found de-novo signatures’ coefficients across all samples. **c** Violin plots of signature-D’s coefficients with respect to block-year (after vs. before 1992). **d** Violin plots of signature-D’s coefficients with respect to sample groups (control, ER-negative, and ER-positive)

As we and others have shown, FFPE-derived sequencing may have distinct variant signatures collectively [17], and therefore de-novo mutational signature decomposition was conducted based on the classical definition of filtered variants for the entire BBD cohort, leading to four different mutational signatures shown in Fig. 3a: two of the observed signatures were primarily enriched for “C>T” paraffin artifacts and highly similar to FFPE/chemistry signatures, which we previously identified in paired comparisons between matched frozen and FFPE samples [17]. One of the de-novo signatures (Signature-D) was found to be highly correlated with collection age of FFPE block (Fig. 3b, c). However, no statistically significant difference was found between this block-year associated signature with cancer status (Fig. 3d). Nonetheless, this highlights the necessity of strict global variant quality control measures beyond variant-level checks for FFPE sequencing data. Furthermore, we assessed a previously published BBD signature that was associated with risk of triple negative BC for association in our dataset [15]; we did not observe presence of the signature in our sample, overall, or within ER+, ER−, or triple negative cases (p > 0.05, data not shown).

### Immunohistochemistry analysis

**Fig. 4:**
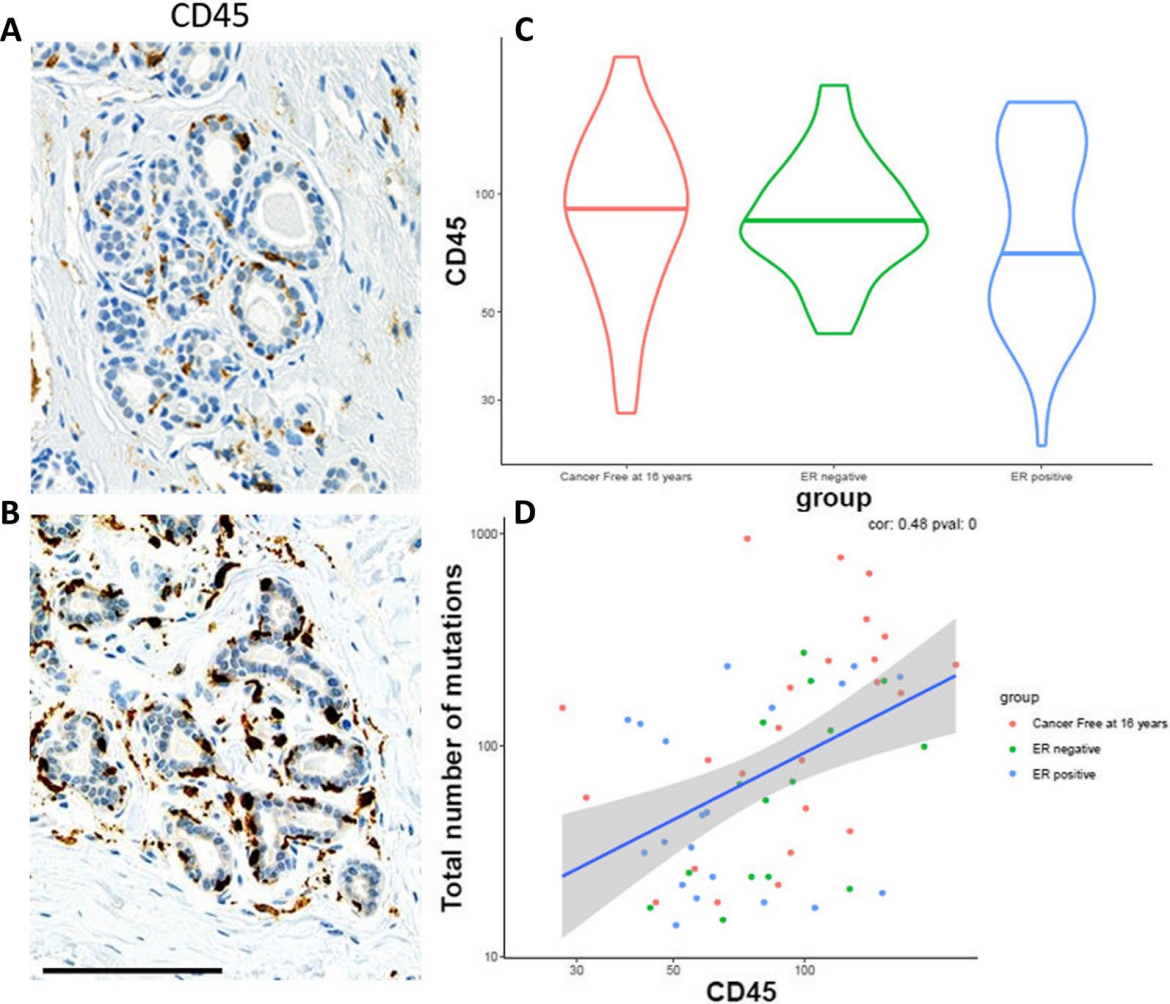
Expression of CD45 by group and mutational burden. CD45 is presented as an H-score. **a** Example staining of low CD45 (18.62). Scale is 100 um. **b** Example staining of high CD45 (60.15). Scale is 100 um. **c** CD45 H score by group. **d** CD45 H score by mutational burden (classic AAF filtering)

To follow-up on the findings that overall mutation was higher among BBD patients who remained cancer-free, we sought to investigate a potential hypothesis, where reduced mutational diversity is associated with (1) increased proliferation, or (2) reduced immune response. To investigate these hypotheses, we performed immunohistochemistry (IHC) analysis of Ki67 (as a marker of proliferation) and CD45 (as a marker of immune response) in normal lobules. Ki67 expression in normal lobules was very low, so analyses were not pursued further. However, CD45 expression was lower in BBD cases as compared to controls (p = 0.19), although not statistically significant, and was positively associated with mutational burden (r = 0.48, p < 0.001), most strongly in controls (r = 0.56, p = 0.004; Fig. 4a–d).

### Germline mutation information

Germline DNA was not available for the vast majority of subjects; however, a subset of 14 patients did have prior germline sequencing data available from other sources. Among these 14, 12 had no pathogenic mutations in pre-disposition genes. One BBD-ER− subject had a pathogenic mutation in *BRCA1* that was also verified with clinical germline testing, and that *BRCA1* mutation was also detected in the BBD tissue in this study. One BBD-ER+ subject had a pathogenic mutation in *BLM* but did not undergo confirmatory clinical germline testing, and the *BLM* mutation was not detected in the subject’s BBD tissue in this study.

## Discussion

In this study, 120 individuals diagnosed with BBD were studied to examine the association of somatic genetic variation with subsequent cancer development. The BBD controls with at least 16 years of cancer-free follow up were compared with BBD patients who developed ER+ or ER− invasive BC within 16 years, with comparison also between the two cancer groups. With a relatively balanced distribution of future cancer status, several genes were shown to have consistent associations with development of BC using 12 statistical methods, including SKAT-O and logistic regression in classical, liberal and strict variant QC schema, although these results were not statistically significant in permutation testing. However, marginally significantly skewed gene-level associations towards lower mutational burden were seen in the overall case population and in ER− cases, supporting the stratified analysis for studying BC risk. Our extensive variant-level and mutational signature-based quality control assessments also highlight the challenges for analyzing biopsy-based BBD archival collections.

Other BBD sequencing-based studies have reported no significant mutations in original BBD tissues, and little to no overlap in the subsequent invasive BC [13–15]. There are multiple possible reasons for the differences in results compared to our study. Each of these studies used surrogates of normal genomes to filter the data; in one study, tissue adjacent to the BBD lesion was used [13], which may inadequately account for field effects and lead to over-filtering. Rather, we used population germline frequencies from published SNP databases to validate our variant filtering and then compared the mutation spectrum between the three groups. Additionally, one prior study focused on a limited number of BBD cases with fibrocystic histology [14], while the other [13] included multiple BBD histological types. Rohan et al. [13] used targeted sequencing based on a capture approach, with substantially lower coverage than the amplicon-based approach here (reported mean variant coverage of 90.4 × compared to mean UMT coverage of 595.6 × and mean depth of 9701.3x), and there was little overlap among the genes we targeted; among our top 10 genes, only *MED12*, *TGFB1*, and *GATA3* were assessed in their study; although not statistically significant, *MED12* and *TGFB1* both had higher number of mutations in controls, and data for *GATA3* was not shown. Soysal et al. [14] reported that two “Tier 2” mutations (with features suggestive of fixation artefacts) in *MAP3K1* and *PIK3CA*, were present in BBD as well as in the subsequent BC. They also reported three *GATA3* mutations (one “likely genuine" and two Tier 2) that were found in tumors but not the fibrocystic lesions; we also identified *GATA3* as a top gene in this study. A third study reported 957 mutations shared between ten invasive BC and the prior BBD lesions [15], as well as a de-novo mutational signature associated with future cancer risk; however, this was not reproduced in our study due possibly to the limited scope of our sequencing panel (93 genes vs. whole-exome).

It is notable that mutations were more common in benign breast tissues of women who did not subsequently develop cancer. Many of the top 10 genes from our study are known to regulate cellular integrity, with half being involved in DNA repair (*BRIP1*, *FAM175A*), and particularly mismatch repair (*MLH1*, *MSH2*, *PMS1*). Regarding possible explanations for more common mutations in controls compared to cases, we hypothesize that cells with more DNA damage might induce an immune-mediated response that protects against cancer development. In support of this, it has been shown that CD45+ cells are present in normal breast tissue [29], are more abundant in BBD compared to normal breast tissues, and B cells are less frequent in BBD cases compared to controls[30]. In this study, CD45 expression was significantly higher with higher mutational burden and was somewhat lower in this series of BBD cases compared to controls, albeit not reaching statistical significance. There is a growing literature on somatic mutations in normal and benign tissues [31–33] leading to questions about the role that somatic mutation and clonal expansion may play in aging and disease, and it has been proffered that analyses of normal and precursor lesions are needed to understand how these may contribute, if at all, to cancer and other disease development [33]. Interestingly, these sequencing studies of normal tissues find mutations in many samples that do not appear to be destined for cancer, and varying hypotheses have been advanced including immune system involvement and tissue architecture [31]. Supporting an immune-mediated suppression of carcinogenesis, lower variant allele frequencies were noted in gene mutations with in-silico predicted neoantigens [31]. If such neoantigens result in mutant proteins, they may elicit an immune-mediated response; thus examining gene expression as well as the presence of immune mediator cells in tissue sections would be good next steps. Many of the significantly associated genes in our study are involved in DNA repair, with potential for recruiting an immune response to clear cells with DNA damage. Whether the increased mutational load results in changes more likely to be immunogenic in benign tissues is intriguing and is an interesting avenue for future research.

Strengths of this study include use of archival tissues from an annotated cohort with long follow-up for cancer outcomes, as well as careful quality control in study design, methods, and the analytical plan. Close attention was paid to handling of these data, including evaluating variant allele frequency differences depending on the nucleotide substitution, combined with multiple approaches to statistical analyses, and defining quality control criteria based on expected variation differences between matched FPPE and fresh frozen samples. In addition to defining top genes by statistical significance, we added additional rigor by requiring significance across multiple analytical approaches. Limitations include the use of older FFPE tissue which is more prone to sequencing artifacts; molecular preservation in such tissues often poses challenges for analysis, particularly for older samples when reagents and processing protocol were less standardized in clinical practice. However, while fresh or frozen tissue is more optimal for sequencing studies, noting the high concordance of single nucleotide variants (SNVs) with publicly available data lends confidence to our careful filtering approach. Furthermore, this study did not include germline sequencing data for filtering of potential somatic mutations in the benign tissue, nor paired sequencing with the subsequent tumor. Finally, due to a small sample size per group, power was limited to detect mutational differences, particularly for individual variants.

In summary, we identified genes suggestive of association with later cancer status, commonly involving DNA repair pathways, and interestingly the variant burden was higher in controls than in either future ER+ or ER− cases. CD45 expression was associated with mutational burden, suggesting a possible role of immunosurveillance in impeding cancer development. Key gene-level findings warrant future validation in large cohort studies, and analyses in paired benign and subsequent tumor tissue.

## Conclusions

Somatic mutations were more frequent in benign breast tissue from women who did not develop cancer, opening questions of clonal diversity or immune-mediated restraint on future cancer development. CD45 expression was positively associated with mutational burden, most strongly in controls. Further studies in both normal and premalignant tissues are needed to better understand the role of somatic gene mutations and their contribution to future cancer development.

## Supplementary Information

**Additional file 1. Fig. S1**: Sample-level QC scatterplot according to genotype call rate and mean UMT coverage.

**Additional file 2. Fig. S2**: Correlation histograms of replicate samples (cyan color) and other un-related samples (red color). Vertical dash-line is at correlation coefficient value of 0.85, for which none of replicate pairs fall below.

**Additional file 3. Table S1**: Meta-data table for all study samples and quality control samples including total number of variants, mean coverage, genotype call rate, and clinical covariates.

**Additional file 4. Fig. S3**: False discovery plot based on four paired fresh-frozen and FFPE samples. False discovery rate was calculated separately for seven mutation categories (C>A, C>G, C>T, C>T at CpG, T>A, T>C & T>G).

**Additional file 5. Fig. S4**: Heatmap and sorted bar plot of cosine similarities between de-novo mutational signatures (A-D) and previously known mutational signatures (COSMIC signatures, QGR signature and FFPE signature).

**Additional file 6. Table S2**: Estimates for the de-novo signatures A-D and previously estimated QGR and FFPE signatures by mutation category.

**Additional file 7. Table S3**: Gene-level results for case-control comparisons for all 12 combinations of QC filters and analysis methods.

**Additional file 8. Table S4**: Gene-level results for ER+ vs. Control comparisons for all 12 combinations of QC filters and analysis methods.

**Additional file 9. Table S5**: Gene-level results for ER- vs. Control comparisons for all 12 combinations of QC filters and analysis methods.

**Additional file 10. Fig. S5**: Variant allele frequency concordances between population frequency from public databases (x-axis) and this studied BBD cohort (y-axis), stratified by SNV substitution categories (e.g. C>A).

**Additional file 11. Fig. S6**: Volcano plots of logistic regression-based odds-ratio (OR) and statistical significance, for ER-positive cases v. controls (A), and ER-negative cases v. controls (B).

## Data Availability

The datasets supporting the conclusions of this article are included within the article and its additional files. The sequencing data generated during the current study are available in the NIH Sequencing Read Archive (SRA) repository, accession number PRJNA734808 (https://www.ncbi.nlm.nih.gov/bioproject/PRJNA734808).

## Abbreviations

BBD: Benign breast disease
BC: Breast cancer
DNA: Deoxyribonucleic acid
FFPE: Formalin fixed, paraffin-embedded
SNP: Single nucleotide polymorphisms
VAF: Variant allele frequency
ER+: Estrogen receptor positive
ER−: Estrogen receptor negative
QC: Quality control
IHC: Immunohistochemistry
SNVs: Single nucleotide variants
MT: Molecular tag
FDR: False discovery rate
AAF: Alternate allele frequency
NMF: Non-negative matrix factorization
gnomAD: Genome Aggregation Database
TOPMED: Trans-Omics for Precision Medicine
IHC: Immunohistochemistry
UMT: Unique Molecular Tag
QGR: QIAGEN GeneRead DNA FFPE Kit
