# Supplementary figures and images for: Somatic mutations in benign breast disease tissues and association with breast cancer risk
Source: BMC Med Genomics. 2021 Jul 14;14:185. doi: 10.1186/s12920-021-01032-8 (PMC8278587; doi:10.1186/s12920-021-01032-8)

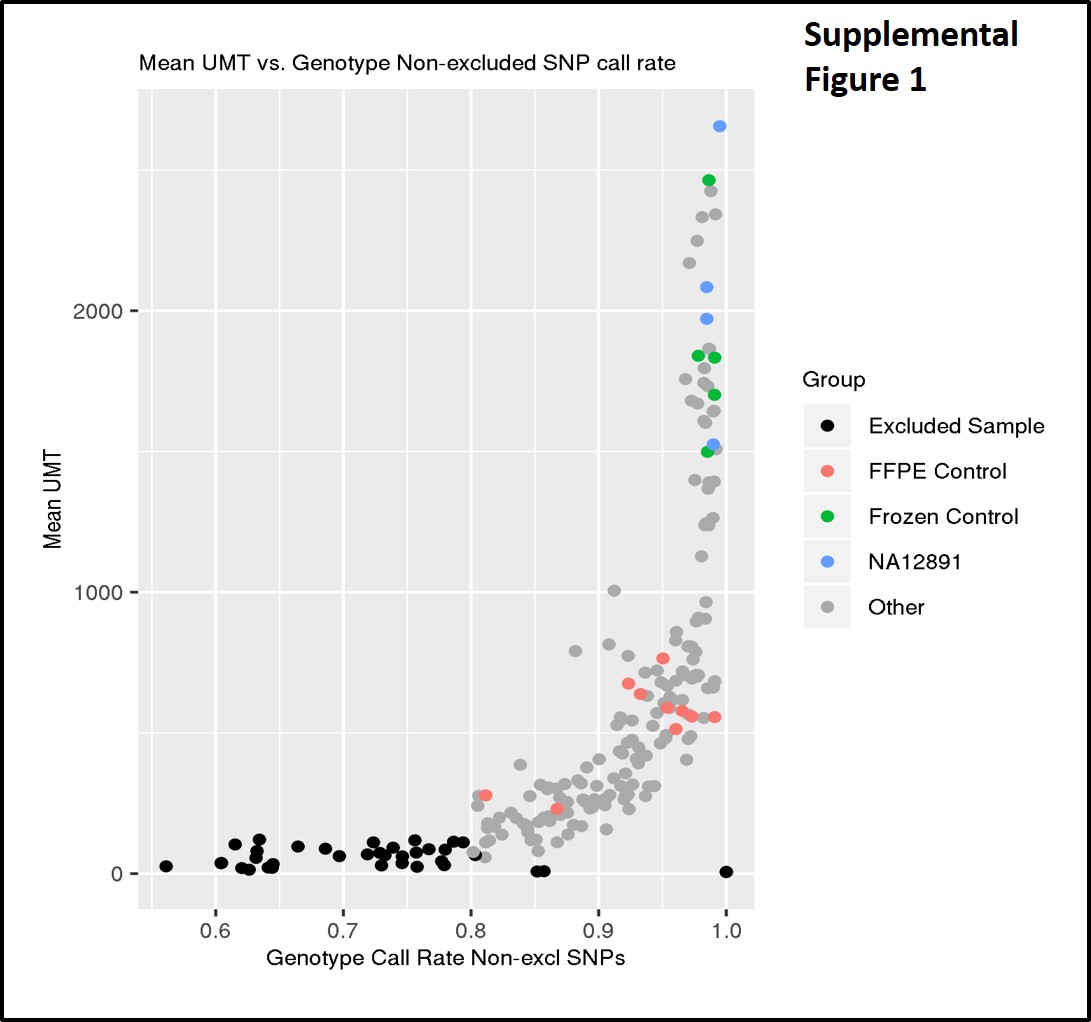

Supplement: Supplementary file 1 — Additional file 1. Fig. S1: Sample-level QC scatterplot according to genotype call rate and mean UMT coverage. [file 12920_2021_1032_MOESM1_ESM.jpg]

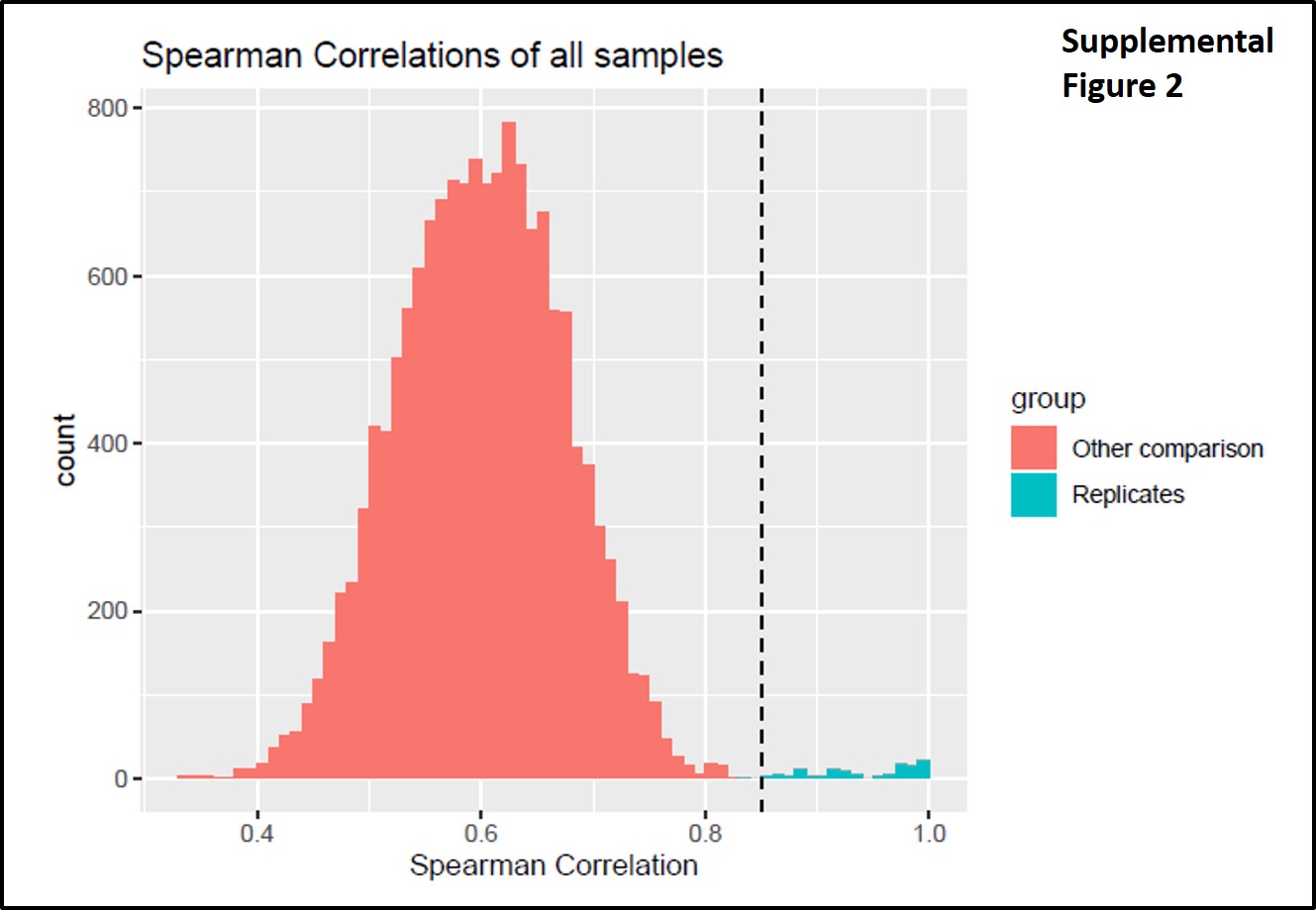

Supplement: Supplementary file 2 — Additional file 2. Fig. S2: Correlation histograms of replicate samples (cyan color) and other un-related samples (red color). Vertical dash-line is at correlation coefficient value of 0.85, for which none of replicate pairs fall below. [file 12920_2021_1032_MOESM2_ESM.jpg]

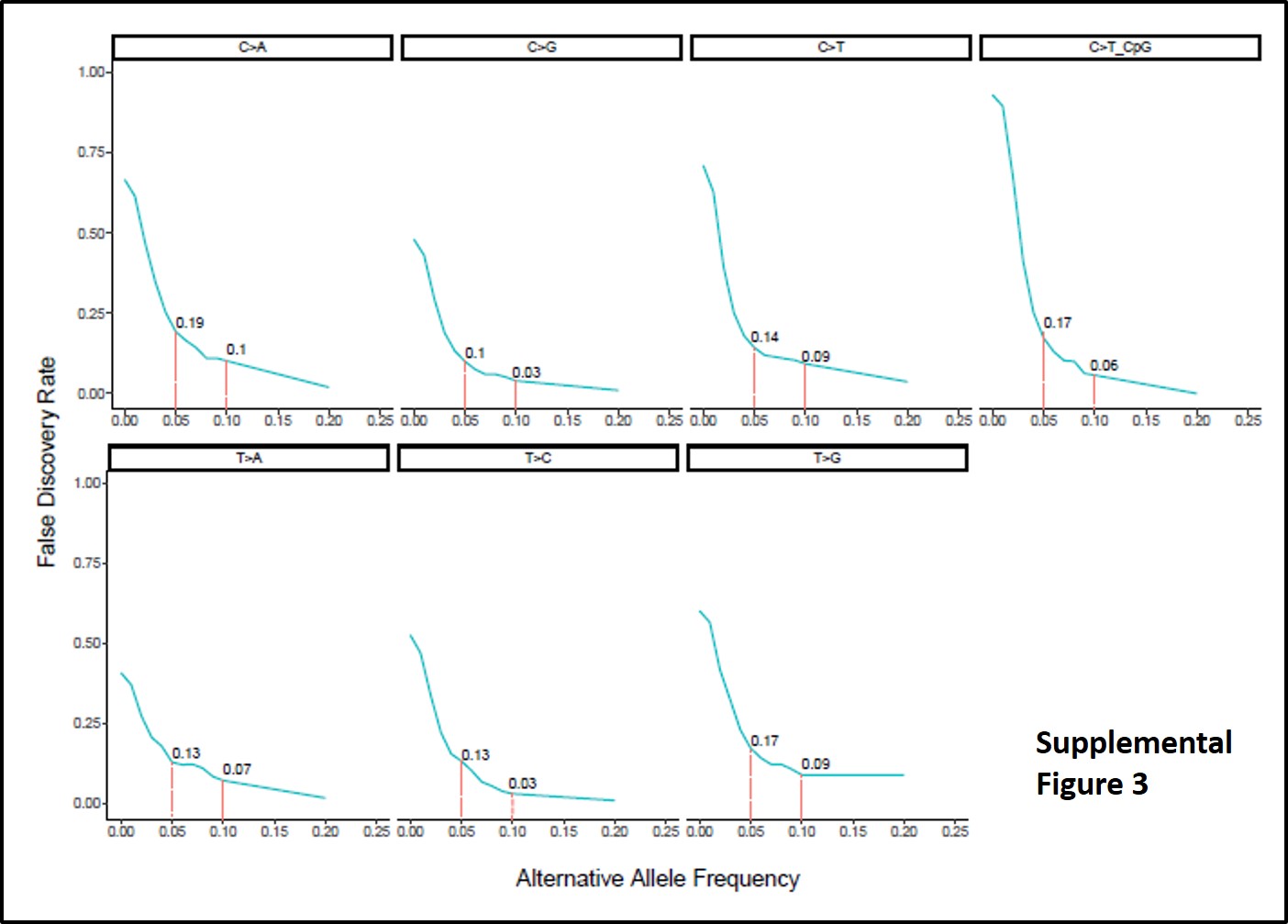

Supplement: Supplementary file 4 — Additional file 4. Fig. S3: False discovery plot based on four paired fresh-frozen and FFPE samples. False discovery rate was calculated separately for seven mutation categories (C>A, C>G, C>T, C>T at CpG, T>A, T>C & T>G). [file 12920_2021_1032_MOESM4_ESM.jpg]

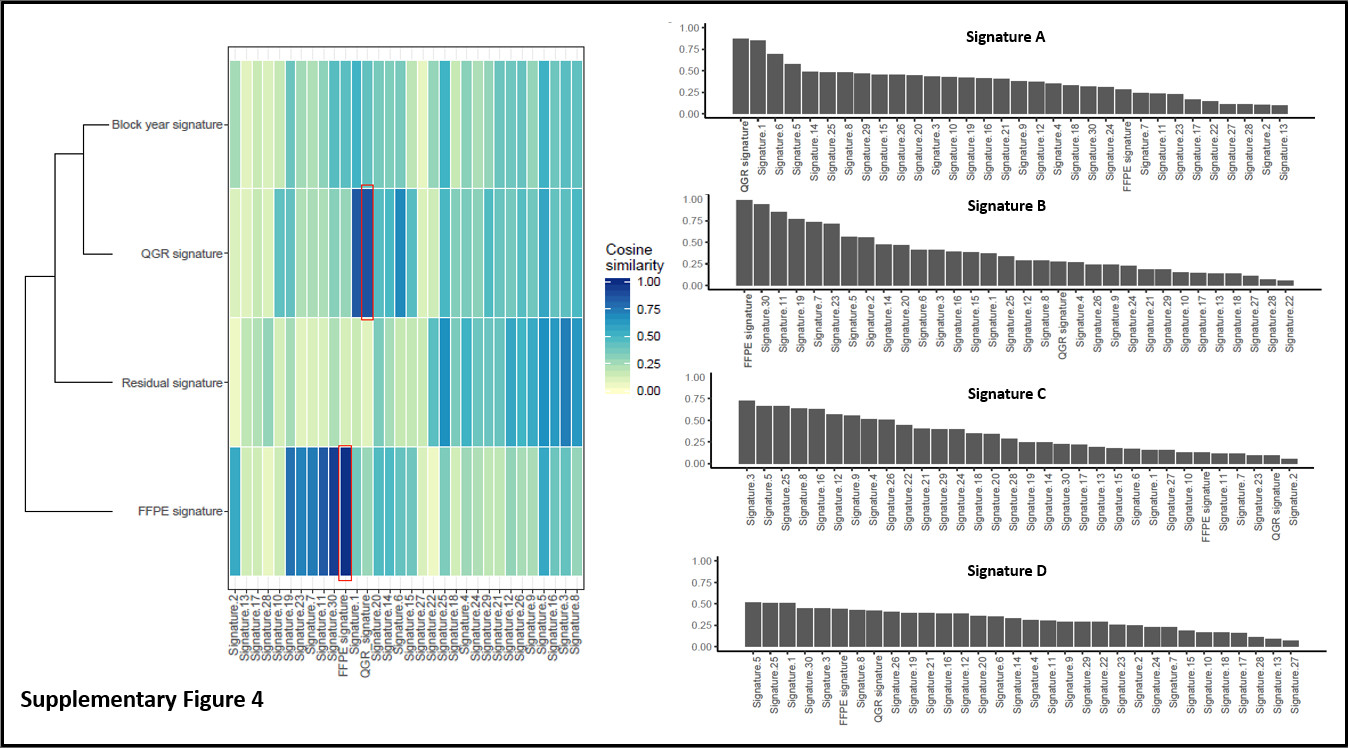

Supplement: Supplementary file 5 — Additional file 5. Fig. S4: Heatmap and sorted bar plot of cosine similarities between de-novo mutational signatures (A-D) and previously known mutational signatures (COSMIC signatures, QGR signature and FFPE signature). [file 12920_2021_1032_MOESM5_ESM.jpg]

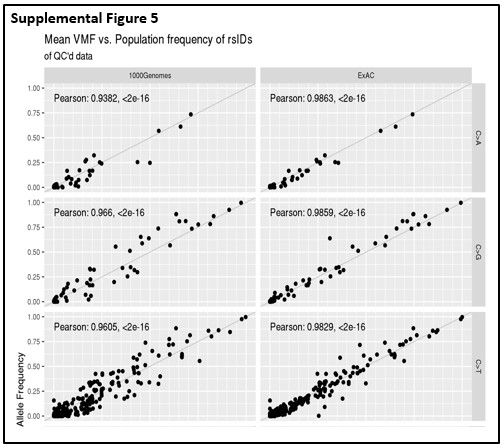

Supplement: Supplementary file 10 — Additional file 10. Fig. S5: Variant allele frequency concordances between population frequency from public databases (x-axis) and this studied BBD cohort (y-axis), stratified by SNV substitution categories (e.g. C>A). [file 12920_2021_1032_MOESM10_ESM.jpg]

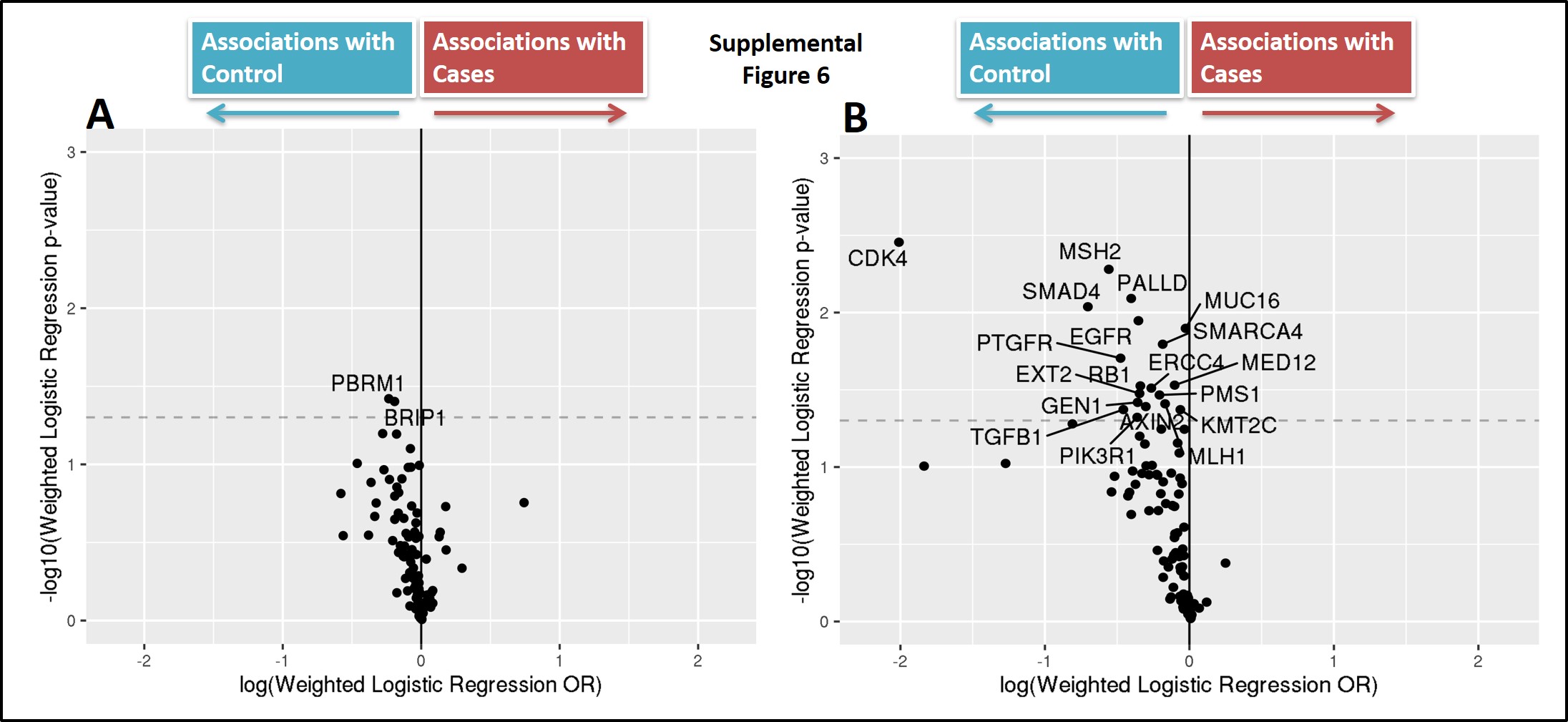

Supplement: Supplementary file 11 — Additional file 11. Fig. S6: Volcano plots of logistic regression-based odds-ratio (OR) and statistical significance, for ER-positive cases v. controls (A), and ER-negative cases v. controls (B). [file 12920_2021_1032_MOESM11_ESM.jpg]
